# Advancements and Applications of Artificial Intelligence in Hypertrophic Cardiomyopathy: A Comprehensive Review

**DOI:** 10.31083/RCM44449

**Published:** 2026-03-12

**Authors:** Huanhuan Ma, Jing Li, Shengjun Ta, Liang Yu, Fangqi Ruan, Liwen Liu

**Affiliations:** ^1^Department of Ultrasound, Xijing Hypertrophic Cardiomyopathy Center, Xijing Hospital, Fourth Military Medical University, 710032 Xi’an, Shaanxi, China; ^2^Office of Graduate Student Affairs, Xi’an Medical University, 710021 Xi’an, Shaanxi, China; ^3^Department of Information, Xijing Hospital, Fourth Military Medical University, 710032 Xi’an, Shaanxi, China

**Keywords:** hypertrophic cardiomyopathy, artificial intelligence, imaging, electrocardiography, genes, clinical management

## Abstract

Hypertrophic cardiomyopathy (HCM) is a common cardiovascular disease and one of the leading causes of exercise-induced sudden cardiac death in adolescents. HCM presents complex diagnostic, prognostic, and management challenges due to the phenotypic heterogeneity and clinical course. Artificial intelligence (AI), machine learning (ML), and deep learning (DL) technologies are expected to transform the roles of echocardiography, electrocardiography (ECG), and cardiac magnetic resonance (CMR) imaging in the clinical management of HCM. AI methods can fully integrate clinical and imaging data to enable a comprehensive assessment of the risk profile of a patient. However, challenges remain, such as insufficient data standardization across multiple sources, limited model interpretability, and data privacy issues. Despite these challenges, AI-based approaches have the potential to revolutionize the management of HCM by providing timely, accurate diagnoses and personalized treatment strategies based on individual patient risk profiles. This review systematically examines the current landscape of AI applications in HCM data analytics, with a focus on methodological advancements and clinical implementations. Furthermore, this review aims to facilitate the transition from experience-based to data-driven paradigms in HCM diagnosis, thereby advancing precision medicine and individualized patient management.

## 1. Introduction

Cardiovascular diseases (CVDs) are the leading cause of premature mortality and disability worldwide. According to the World Health Organisation (WHO), approximately 17.9 million people died of CVDs in 2019, accounting for 32% of global deaths [1]. This high morbidity and lethality not only cause great suffering to patients and their families but also impose substantial global healthcare costs and economic burdens. Among CVDs, hypertrophic cardiomyopathy (HCM) warrants particular clinical attention due to its distinctive myocardial hypertrophy and high risk of sudden cardiac death (SCD).

Originally deemed a rare genetic cardiac disorder with diagnostic challenges and limited therapies, HCM is now recognized as much more common with worldwide distribution, with a prevalence of 1:500–1:200 in adults [2]. The etiology of HCM is complex, with significant genetic heterogeneity, as approximately 50–70% of HCM cases are attributable to pathogenic variants in sarcomeric genes [3]. The clinical manifestations of HCM are diverse, with significant prognostic heterogeneity: some individuals remain asymptomatic lifelong, while others develop severe complications including SCD, left ventricular outflow tract obstruction (LVOTO), and atrial fibrillation (AF) [4]. HCM’s heterogeneous clinical characteristics result in a multidimensional and intricate decision-making process, which involves a large amount of complex data from imaging, dynamic electrophysiology, and genomics. This complexity makes it urgent for clinicians to accurately analyze such multifaceted information from multiple domains and translate it into practical clinical decisions. In addition, as patients’ health awareness continues to rise, their expectations for healthcare services are gradually shifting towards greater efficiency and personalization. In short, doctors are faced with the challenge of interpreting massive amounts of data with their professional knowledge, as well as optimizing the process and improving efficiency throughout the treatment process, to adapt to the development of medicine and meet the diverse needs of patients [5, 6].

In recent years, the rapid development of artificial intelligence (AI) technology has offered novel perspectives for HCM clinical management. AI techniques can extract key features from huge amounts of medical data and build efficient medical models. These models cover medical aspects from disease diagnosis to prognosis assessment, significantly improving the accuracy and efficiency of HCM management. For example, deep learning (DL) networks have enabled both echocardiographic view recognition and automatic detection of HCM [7]; Furthermore, they demonstrate great potential in accurately identifying HCM-related abnormal features in electrocardiography (ECG) [8]; Additionally, the innovative combination of machine learning (ML) models with clinical cardiac magnetic resonance (CMR) features, by constructing nonlinear models, can more accurately predict dee in HCM patients [9]. Furthermore, these ML models enhance HCM patient risk assessment by predicting arrhythmic events that may lead to SCD and identifying patients at risk of developing AF and heart failure (HF). Indeed, modern medicine faces a deluge of data that is beyond human comprehension and analysis without the aid of AI and ML.

This review aims to systematically explore the current status, challenges, and future directions of AI applications in HCM management, thereby providing a foundation for further research and clinical practice. By conducting an in-depth analysis of the central role of AI in the clinical management of HCM, it seeks to provide novel insights for advancing precision medicine in this condition.

## 2. Artificial Intelligence Overview

AI refers to the capability of machines to perform tasks that typically require human intelligence, such as image recognition, planning, language comprehension, and voice recognition [10]. In practice, AI enables machines to achieve autonomous decision-making using collected data. Within medicine, this typically involves leveraging clinical data (e.g., health records, imaging data) to predict diagnoses, identify novel disease patterns, or determine optimal treatment options. Following its inception in the mid-20th century, AI development was initially constrained by computational limitations. However, advances over the past 25 years, fueled by exponentially growing datasets, have driven continuous progress in the field. The advent of DL architectures and large-scale datasets in recent years has revolutionized AI applications in healthcare [11].

ML is a branch of AI that focuses on developing algorithms and models to train computers in analyzing data and making predictions [12]. ML learn by continuously processing data, thereby refining its predictive capabilities. Utilizes deep neural networks (DNN) composed of multiple stacked layers. Inspired by the human brain’s capacity for abstract feature extraction, DL employs these hierarchical layers to progressively extract features and learn complex representations from data (such as complex medical data), enabling sophisticated pattern recognition and prediction tasks. This process relies on the backpropagation algorithm to uncover complex structures in data, enabling intelligent medical diagnostic models to adjust their model parameters based on prior diagnostic outcomes, thereby enhancing their ability to recognize disease features [13]. Crucially, by forming high-level abstract representations through the combination of low-level features, DL allows computers to comprehend complex data without relying on manual prior knowledge.

The AI algorithms integrating different examination methods (Table 1, Ref. [7, 8, 9, 14, 15, 16, 17, 18, 19, 20, 21, 22, 23, 24, 25, 26, 27, 28, 29, 30, 31, 32, 33, 34, 35, 36, 37]) can not only automate repetitive operations but also improve the accuracy of diagnosis and prognostic assessment for HCM, providing support for precision medicine in diagnosis and management.

**Table 1.** **Summary of reviewed studies on artificial intelligence (AI) methods applied to the medical data of hypertrophic cardiomyopathy (HCM), including their performance and characteristics**.

Model outcome	Data input	Sample size	AI method	Model performance	Reference
Detection of diseases: HCM, DCM	Echocardiography image data	60	BPNN, SVM, K-NN	Accuracy: 90.2	Balaji *et al*. [14]
Detection of HCM	Echocardiography image data	1553	SlowFast, I3D	Accuracy: 95.28	Almadani *et al*. [15]
Detection of diseases: HCM, ASD, DCM, prior MI	Echocardiography image data	2189	CNN (AIEchoDx)	AUC: 99.57 (HCM)	Liu *et al*. [16]
				AUC: 99.50 (ASD)	
				AUC: 98.75 (DCM)	
				AUC: 98.52 (prior MI)	
Detection of diseases: HCM, CA, PAH	Echocardiography image data	6793	CNN	C statistics: 0.93 (HCM)	Zhang *et al*. [7]
				C statistics: 0.87 (CA)	
				C statistics: 0.85 (PAH)	
Detecting the presence of myocardial scarring in patients with HCM	CMR image data	859	CNN, FCN	internal dataset AUC: 0.83	Fahmy *et al*. [17]
				external dataset AUC: 0.74	
VNE images were substituted for LGE to assess HCM myocardial tissue characteristics and lesions	CMR image data	1348	CNN, GAN	hyperintensity myocardial lesions: r = 0.77–0.79/ICC = 0.77–0.87	Zhang *et al*. [18]
				intermediate-intensity lesions: r = 0.70–0.76/ICC = 0.82–0.85	
Segmentation and quantification of myocardial scarring in LGE images of HCM patients	CMR image data	191	CNN	Accuracy: 89	Fahmy *et al*. [19]
Detection of diseases: HCM, DCM	CMR image data	1200	CNN	Accuracy: 0.982 ± 0.009	Germain *et al*. [20]
Detection of HCM	12-leads ECG (Raw data)	3060	CNN	AUC: 0.96	Ko *et al*. [8]
Detection of HCM in children and adolescents	12-leads ECG (Raw data)	300	CNN	AUC: 0.98	Siontis *et al*. [21]
Detection of HCM	12-leads ECG (Raw data)	4640	CNN	AUC: 0.922	Siontis *et al*. [22]
Diagnosis of 15 CVDs, including HCM	12-leads ECG (Raw data)	244,077	CNN (ResNet), XGB	AUC >0.90 (HCM)	Kalmady *et al*. [23]
HF severity classification	12-leads ECG (Raw data)	463	DNN	AUC: 0.71 (mild HF)	Togo *et al*. [24]
				AUC: 0.71 (moderate HF)	
				AUC: 0.80 (severe HF)	
Prediction of genetic mutation risk in HCM patients	CMR image data	198	DeeplabV3+, InceptionResnetV2, LSTM	AUC: 0.84 (The combination of the DL and the Toronto score)	Zhou *et al*. [25]
Predict a positive genotype in patients with HCM	Echocardiography image data	99	DCNN	AUC: 0.86 (Combining Mayo score and DCNN-derived)	Morita *et al*. [26]
				AUC: 0.84 (Combining Toronto score and DCNN-derived)	
Predict a positive genotype in patients with HCM	12-leads ECG (Raw data)	254	CNN	AUC: 0.89	Chen *et al*. [27]
Reveal the genetic factors of HCM	CMR image data, Genotype data	42,194	MSMM	-	Ning *et al*. [28]
Explore the phenotypic differences between HCM subgroups associated with *MYH7* and *MYBPC3*	CMR image data	102	SVM, PCA	AUC: 0.968 (Feature selection dataset)	Wang *et al*. [29]
				AUC: 0.886 (Test validation dataset)	
Differential diagnosis of LVH: CA, HCM	Echocardiography image data	23,745	CNN (DeepLabv3, ResNet3D)	Internal dataset:	Duffy *et al*. [30]
				AUC: 0.98 (HCM)	
				AUC: 0.83 (CA)	
				External dataset:	
				AUC: 0.89 (HCM)	
				AUC: 0.79 (CA)	
Differential diagnosis of LVH: HHD, HCM, CA	Echocardiography image data	930	CNN-LSTM	AUC: 0.962 (HHD)	Hwang *et al*. [31]
				AUC: 0.982 (HCM)	
				AUC: 0.996 (CA)	
Differential diagnosis of LVH: CA, HCM, HHD	CMR image data	355	CNN (2D Res-Unet)	Classification accuracy: 77.4	Diao *et al*. [32]
				Model 3 demonstrated the best performance:	
				AUC: 0.895–0.980 (CA)	
				AUC: 0.879–0.984 (HCM)	
				AUC: 0.848–0.983 (HHD)	
Differential diagnosis of LVH: HCM, Fabry	CMR image data	248	CNN	AUC: 0.914 (Internal dataset)	Chen *et al*. [33]
				AUC: 0.918 (External validation)	
Differential diagnosis of LVH	CMR image data	187	CNN	AUC: 0.830 (DL-myo)	Wang *et al*. [34]
			(ResNet32)	AUC: 0.766 (DL-box)	
				AUC: 0.795 (DL-nomyo models)	
				AUC: 0.545 (Native T1)	
				AUC: 0.800 (Radiomics)	
Discriminate major cardiovascular events in patients with HCM	Clinical features and Echocardiography image data	2111	LR, LDA, RF, SVM	The LR model demonstrated the best performance:	Rhee *et al*. [35]
				AUC: 0.80 (MACE)	
				AUC: 0.789 (All-cause death)	
				AUC: 0.798 (HF-adm)	
				AUC: 0.807 (Stroke)	
Discriminate major cardiovascular events in patients with HCM	Clinical variables, Demographic characteristics, Genetic data	2302	RF, XGBoost, SVM	AUC: 0.90 (VT)	Smole *et al*. [36]
				AUC: 0.88 (HF)	
				AUC: 0.87 (ICD)	
Discriminate major cardiovascular events in patients with HCM	CMR image data and clinical characteristic data	758	Light GBM, RFE	AUC: 0.830 (Internal cohort)	Zhao *et al*. [9]
				AUC: 0.812 (The external test cohort)	
Predict new-onset AF in patients with HCM	Clinical variables	1069	LR with Lasso regularization	AUC: 0.84	Lu *et al*. [37]

BPNN, back propagation neural network; SVM, support vector machine; DCM, dilated cardiomyopathy; HCM, hypertrophic cardiomyopathy; I3D, two-stream inflated 3D convNets; ASD, atrial septal defect; prior MI, prior myocardial infarction; CA, cardiac amyloidosis; PAH, pulmonary arterial hypertension; CNN, convolutional neural network; CMR, cardiac magnetic resonance; FCN, fully-connected network; VNE, virtual native enhancement; LGE, late gadolinium enhanced; GAN, generative adversarial network; ECG, electrocardiography; ICC, intraclass correlation coefficients; XGB, extreme gradient boosting; DNN, deep neural network; LSTM, long short-term memory; DCNN, deep convolutional neural network; MSMM, myocardial segmentation and measurement method; PCA, principal component analysis; LVH, left ventricular hypertrophy; HHD, hypertensive heart disease; LR, logistic regression; RF, random forest; LDA, linear discriminant analysis; CVDs, cardiovascular diseases; HF, heart failure; VT, ventricular tachycardia; RFE, recursive feature elimination; Light GBM, light gradient boosting machine; AF, atrial fibrillation; DL, deep learning; MACE, major adverse cardiovascular event; ICD, implantable cardioverter defibrillator; AIEchoDx, AI echocardiogram diagnosis network; K-NN, K-nearest neighbour; AUC, area under the curve.

## 3. Artificial Intelligence in the Medical Data of Hypertrophic Cardiomyopathy

### 3.1 Echocardiography

Echocardiography is the preferred method for diagnosing HCM due to its ease of use and ability to provide real-time information on cardiac structure and function [38]. This imaging modality is essential for assessing left ventricular wall thickness, LVOTO, systolic anterior motion (SAM) of the mitral valve, mitral regurgitation severity, as well as left ventricular diastolic and systolic function [39]. However, the phenotypic heterogeneity of HCM often complicates echocardiographic interpretation, particularly in early or atypical cases. Traditional manual analysis is susceptible to subjectivity, highlighting an urgent need for intelligent tools to enhance diagnostic efficiency.

**Fig. 1.**
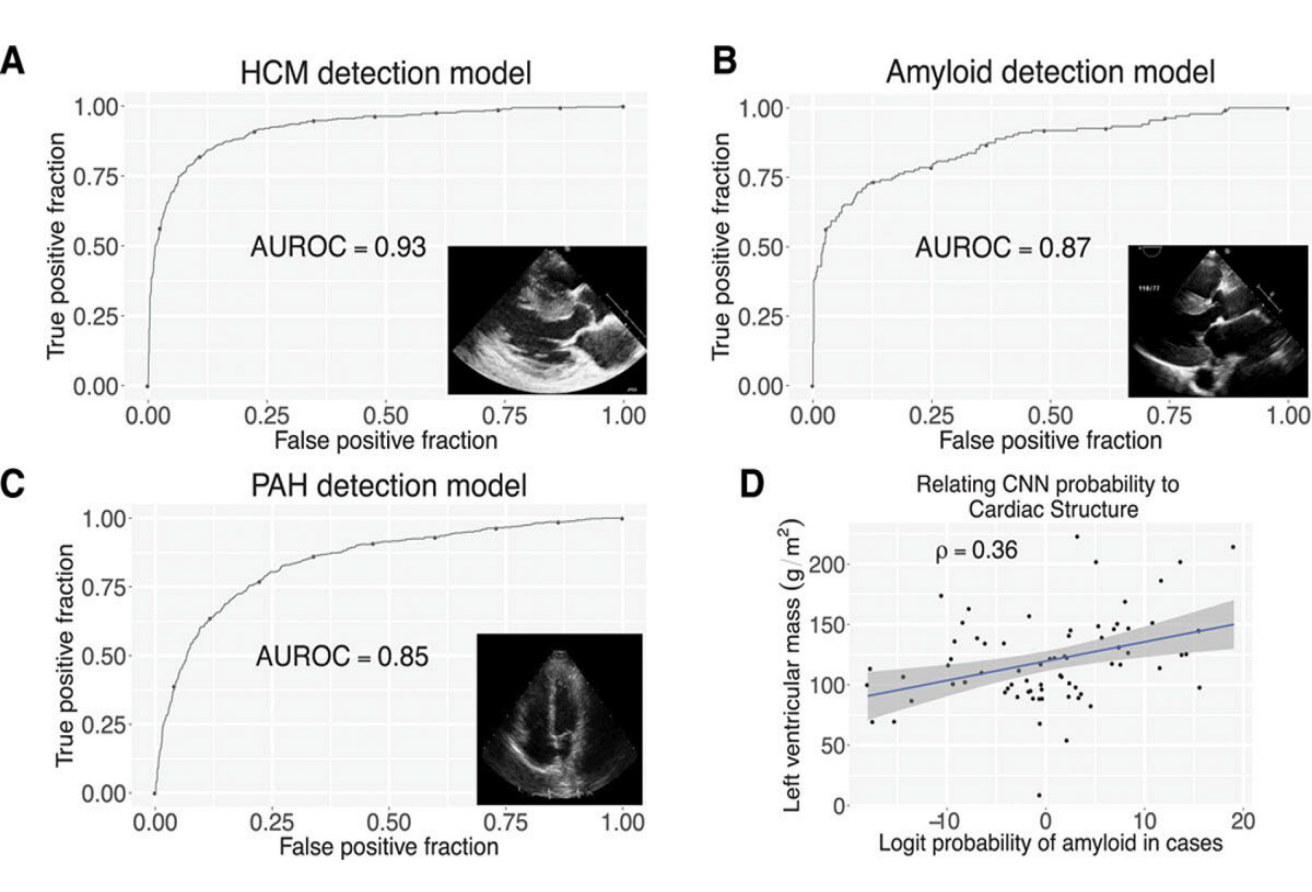
**CNNs enable the detection of abnormal myocardial diseases**. (A) Through (C), the receiver operating characteristic curves for the detection of HCM (A), cardiac amyloid (B), and PAH (C), respectively. (D) depicts the correlation between the probability of amyloidosis and left ventricular mass, where the blue line represents the linear regression fit, and the gray shaded region denotes the 95% confidence interval (CI). AUROC, area under the receiver operating characteristic curve [7].

In this context, AI technology has gradually become a key tool to overcome the limitations of traditional ultrasound diagnostics. Early research focused on single-frame image analysis. In studies exploring automated HCM diagnosis via echocardiography, an ML approach classified hearts as HCM, normal, or dilated cardiomyopathy (DCM), achieving the highest overall accuracy of 90.20% [14]. This result demonstrates the potential of AI in single-frame image analysis. To further improve diagnostic accuracy, the researchers explored the application of multiview echocardiography. Almadani *et al*. [15] developed the HCM-Echo-VAR-Ensemble model, a deep integrated learning framework. This model performed binary classification on echocardiographic videos (HCM vs. non-HCM), achieving a diagnostic accuracy of 95.28% [15]. Although this study improves the sensitivity and accuracy of HCM detection, it focuses only on specific views and does not incorporate other diagnostically valuable perspectives, limiting model comprehensiveness. In a similar study, the researchers developed the AI echocardiogram diagnosis network (AIEchoDx) DL framework, which not only differentiates HCM from other CVDs but also accurately identifies key pathological regions of interest associated with each disease by visualizing the decision-making process [16]. In other research on view recognition, Zhang *et al*. [7] developed a convolutional neural network (CNN) model capable of multiple tasks, trained and evaluated on 14,035 echocardiograms. Their model accurately identified parasternal long-axis views with 96% recognition accuracy and achieved a C-statistic of 0.93 for HCM detection (Fig. 1) [7]. This study further validates the high accuracy of AI in echocardiographic view recognition.

Recent AI-echocardiography integration research has advanced from single-image classification to dynamic time-series analysis, multi-view integration, collaborative methods, and enhanced interpretability. Future work must reduce model dependencies on specific views and limited data diversity. Establishing a cross-modal, multi-center training framework will improve HCM diagnosis, enabling deeper clinical understanding and facilitating personalized treatment strategies.

### 3.2 Cardiac Magnetic Resonance

**Fig. 2.**
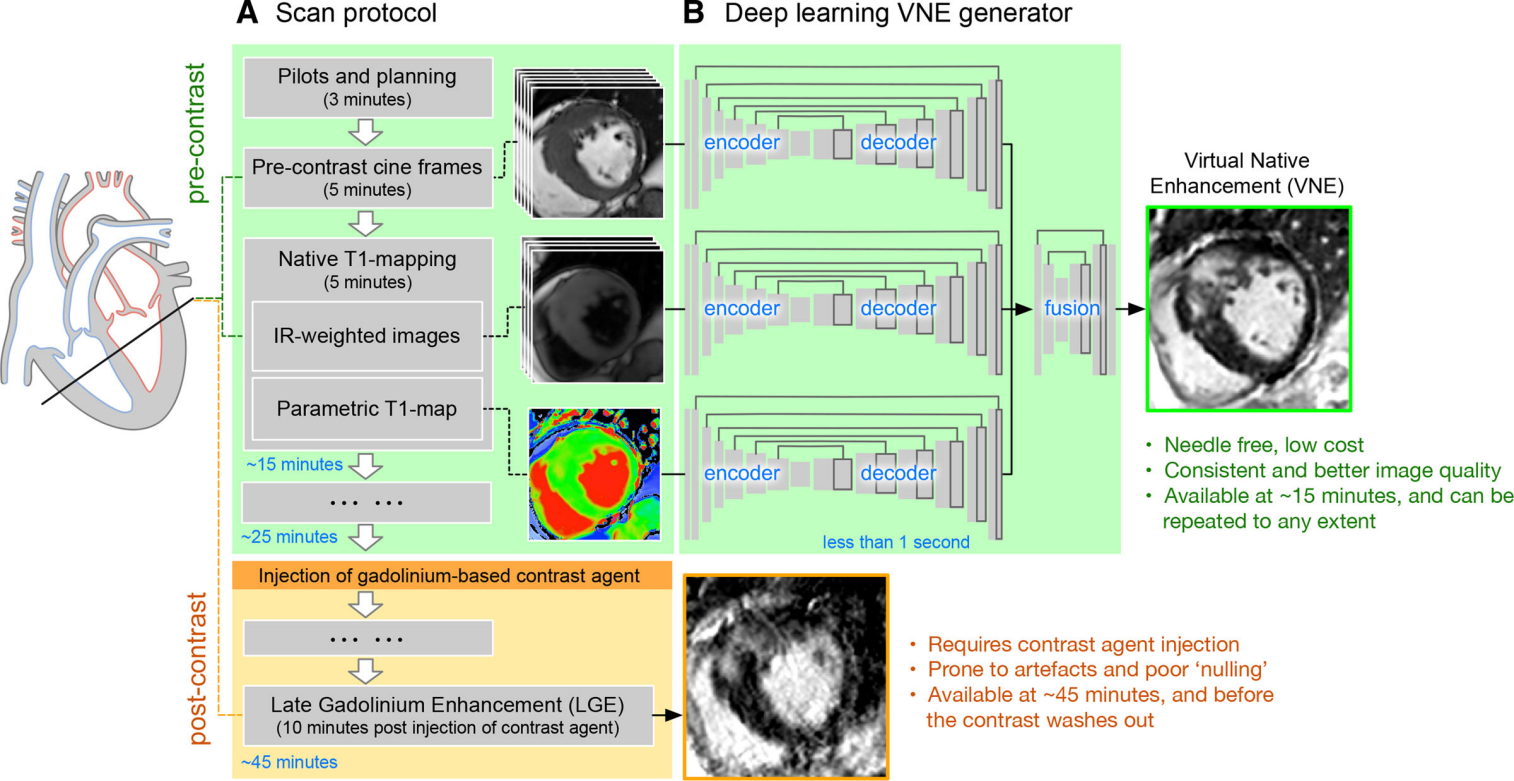
**Overviews VNE imaging technology**. (A) presents a simplified scan protocol for the HCM Registry, covering pre-contrast cine imaging, T1 mapping, and post-contrast late gadolinium enhancement. (B) explains that VNE images are generated by feeding native CMR images into three U-nets to extract feature maps, which are then fused by a neural network block in under 1 second after training. VNE, virtual native enhancement [18].

CMR, the gold standard for non-invasive myocardial function assessment, holds irreplaceable value in HCM management [40]. Beyond precisely quantifying cardiac structure and function, CMR visualizes myocardial fibrosis via gadolinium-enhanced late gadolinium enhancement (LGE), providing critical diagnostic and prognostic insights. However, LGE requires gadolinium-based contrast agents (GBCA), which carry risks like nephrogenic systemic fibrosis. Nearly half of HCM patients undergo repeated GBCA exposure due to absent scarring, driving demand for safer alternatives [17]. To reduce unnecessary GBCA use, Fahmy *et al*. [17] integrated DL with radiomics to build a screening model identifying scarless HCM patients without LGE. This approach outperformed single-technique models in internal/external validation, offering a clinical pre-screening tool. Zhang *et al*.’s [18] DL-based CMR virtual native enhancement (VNE) technique addresses this by generating fibrosis-mimicking images highly consistent with LGE through feature extraction, enabling contrast-free lesion quantification in multicenter data (Fig. 2, Ref. [18]). A limitation is VNE’s dependency on original image quality, requiring validation in low signal-to-noise scenarios. Although radiomics-DL fusion models outperform single-technique approaches in HCM diagnosis, their clinical adoption remains limited relative to practical demands. Advancing precision medicine necessitates continuous architecture refinement, innovative algorithm development, and multimodal data integration to enhance generalizability and strengthen clinical utility.

To address the need for enhanced clinical utility, researchers developed a DL model integrating LGE with cine-MRI. This leverages cine sequences’ high soft-tissue resolution to dynamically define myocardial boundaries. The approach significantly improves quantification accuracy in low-contrast scar regions, achieving clinically acceptable consistency between automated and manual measurements while overcoming the inefficiency and subjectivity of traditional methods [19]. Separately, Germain *et al*. [20] pioneered CNN-based classification of HCM versus other cardiomyopathies using cine-MR images, attaining 98% diagnostic accuracy. This result not only confirms that AI can reproduce the diagnostic logic of human experts but also highlights its efficiency and consistency in multi-disease diagnosis.

Current integration of AI and CMR is advancing precision and safety in HCM management through multidimensional applications such as non-invasive fibrosis assessment and multi-sequence synergy. Future development requires consolidating multimodal clinical data to establish AI-driven analytics, enabling closed-loop support across the diagnostic-therapeutic continuum.

### 3.3 Electrocardiography

ECG remains essential for evaluating cardiovascular symptoms and provides critical diagnostic information in known or suspected HCM cases [41]. Nevertheless, ECG interpretation is expert-dependent, and HCM exhibits non-specific features such as left ventricular hypertrophy and ST-T changes. Recent advances in DL have enhanced HCM diagnostic performance by detecting subtle waveform patterns.

Earlier studies attempted to train models to recognize HCM using ECG indicators but were limited by the performance of traditional ML algorithms. In recent years, several studies have optimized model performance through different algorithms AI-ECG model based on CNN analyzes standard 12-lead ECG signals to achieve efficient diagnosis of HCM. For example, one study used a CNN architecture to extract time-frequency domain features from ECG data of adult HCM. This model achieved 87% sensitivity and 90% specificity in an independent validation set, with an area under the curve (AUC) of 0.96 [8]. The algorithm was further validated in pediatric HCM patients, showing an AUC of 0.98, a sensitivity of 92%, a specificity of 95%, and improving performance with increasing age [21]. It was then externally validated in a multicenter, international case-control study. The results showed that the algorithm had an AUC of 0.92 for subjects, a diagnostic accuracy of 86.9% for HCM, a sensitivity of 82.8%, and a specificity of 87.7% [22]. Although this work demonstrated value in clinical practice and screening situations for HCM detection, the algorithm needs to be evaluated in prospective studies.

A DL prediction model based on residual networks further extends the application scenarios of ECG. This approach simultaneously predicted 15 CVDs, including HCM, achieving an AUC of >90% for those diagnosed with HCM [23]. Moreover, researchers created a deep neural network-based cardiac function classification model for HCM. It automatically classifies the severity of HF (e.g., New York Heart Association (NYHA) classification) in patients with HCM by analyzing 12-lead ECG data to assist in optimizing clinical decision-making [24]. However, this model’s training data primarily came from a single center and did not cover all HCM phenotypic subgroups, which may affect its generalization ability.

In the future, with advancements in AI, the accumulation of more data, and continued validation efforts, DL models are expected to play a more critical role in ECG-based diagnosis of HCM, providing more accurate and efficient support for clinical diagnostic screening. This will ultimately improve the overall diagnosis and treatment of HCM.

## 4. Hypertrophic Cardiomyopathy Genotype Prediction

HCM is an autosomal dominant disease, and its pathogenesis is closely related to mutations in sarcomeric genes (e.g., *MYH7, MYBPC3*, etc.) [42, 43]. Although genetic testing can provide individualized risk assessment and therapeutic guidance for HCM patients, there is significant heterogeneity in the associations between HCM phenotypes and genotypes, which significantly hampers the realization of precision diagnosis and treatment. To address this challenge, AI has emerged as a tool to enable non-invasive prediction of genotype and elucidation of genetic mechanisms by integrating multimodal clinical data and genetic information.

In recent years, AI technologies have advanced non-invasive genotype prediction by integrating imaging data with genetic information. DL models based on CMR cine imaging have significantly enhanced the identification of HCM-causing mutations by integrating imaging features with traditional scoring systems [25]. Morita’s team [26] applied a DL framework to multiview echocardiography (short-axis, long-axis, and apical) and compared its performance with conventional models. Their novel hybrid approach, combining traditional and DCNN-derived models, demonstrated superior accuracy in predicting positive genotype in patients with HCM [26]. Similarly, Chen *et al*. [27] identified genetic markers associated with HCM in 12-lead ECG data of HCM patients. The CNN model they developed achieved an AUC of 0.89 in an external validation set and could guide clinical decision-making via SCD risk stratification [27]. Notably, this model’s training data did not cover all HCM-related mutation types, which may limit its broad applicability.

In the field of genetic mechanism analysis, AI technology has enabled novel approaches to exploring the etiology of HCM through image-genome association studies. For instance, Ning *et al*. [28] employed DL to quantify 12 left ventricular regional wall thicknesses (LVRWT) based on CMR data from the UK Biobank. Subsequent genome-wide association analysis identified 72 genetic loci significantly associated with LVRWT. Mendelian randomization further confirmed a causal association between LVRWT and HCM, providing new insights into the disease’s genetic mechanisms [28]. Additionally, Wang *et al*. [29] developed a radiomics-ML fusion model that, when using the support vector machine (SVM) model combined with principal component analysis (PCA), achieved an accuracy of 92.0% and AUC of 0.968 in distinguishing *MYH7* and *MYBPC3* mutation carriers based on CMR features.

However, currently no AI models forecast phenotype development, yet exploring this capability could significantly enhance understanding and follow-up for individuals carrying mutations.

## 5. Hypertrophic Cardiomyopathy and Other Left Ventricular Hypertrophy Disease Identification

Left ventricular hypertrophy (LVH) is a common phenotype of HCM, hypertensive heart disease (HHD), cardiac amyloidosis (CA), and other diseases [44]. Identifying its underlying etiology is critical for guiding treatment strategies and prognostic assessment [45]. AI is overcoming limitations of traditional diagnostic approaches by significantly enhancing the efficiency and accuracy of LVH etiologic differentiation through the integration of multimodal imaging data and dynamic feature analysis.

In the field of echocardiography, AI technology has significantly improved diagnostic performance through view standardization and multidimensional feature fusion, and Duffy’s team [30] has developed a multicenter-based DL model that can automatically measure left ventricle parameters and differentiate HCM from other causes. The model accurately assesses left ventricle wall thickness and distinguishes HCM from other etiologies of LVH [30]. However, it did not incorporate a specific view of apical HCM, limiting its ability to identify atypical cases. In a follow-up study, a hybrid convolutional-long and short-term memory network (CNN-LSTM) was introduced, improving the overall diagnostic accuracy for HCM, HHD, and CA to 92.3% by analyzing the temporal dynamics of the parasternal long-axis, short-axis, and apical multi-chamber views. However, external validation was only performed at a single center, and its ability to generalize across centers still requires verification [31].

In the field of CMR, Diao *et al*. [32] developed a multi-channel DL model to automatically diagnose the cause of LVH by extracting features from multiple sequences of CMR images. The model’s diagnostic accuracy in discriminating HCM, CA, and HHD was not inferior to that of radiologists [32]. Chen *et al*. [33] proposed the DL model “MRI short-axis LV hypertrophy classifier (MSLVHC)”, which specifically differentiates between HCM and Fabry cardiomyopathy. In internal and external analyses, it attained an AUC of 0.914 and 0.918, respectively, demonstrating diagnostic efficacy comparable to radiologists [33]. Meanwhile, a study confirms that DL models based on CMR T1 mapping significantly outperform traditional imaging genomics methods in discriminating HCM and HHD, highlighting the unique advantages of AI in mining quantitative imaging markers [34].

Currently, AI has progressed from automated parameter measurement to complex etiological classification, leveraging view standardization and multi-sequence synergy. However, its clinical translation remains constrained by insufficient diversity in training data and a reliance on high-quality images. Moving forward, advancing LVH differential diagnosis toward intelligence-driven precision will necessitate cross-agency collaboration and low-quality data augmentation techniques.

## 6. Hypertrophic Cardiomyopathy Risk Stratification and Prognosis

HCM, as the most common genetically inherited CVDs, is characterized by a high risk of sudden death. Approximately 10–15% of HCM patients experience major adverse cardiovascular events (MACEs) annually [46, 47]. Although the HCM Risk-SCD model recommended by current guidelines predicts the risk of SCD and guides implantable cardioverter defibrillator (ICD) use, its predictive scope does not encompass a broader range of MACEs, and its accuracy has limitations.

With the increasing application of ML in medical research, in an HCM risk stratification prediction study, Rhee’s team [35] incorporated clinical and echocardiographic features of HCM patients into an ML model to achieve accurate stratification of MACEs (all-cause death, HF, and stroke). This research leverages AI and ML to investigate the critical role of diverse clinical and imaging markers in MACEs occurrence among HCM patients. Furthermore, Smole *et al*. [36] developed an integrated predictive model incorporating demographic, genetic, and multi-modal imaging data, which achieved the highest predictive accuracy for ventricular tachycardia (VT), HF, and ICD activation, with AUC values of 0.90, 0.88, and 0.87, respectively. Recently, Zhao *et al*. [9] innovatively developed and validated an ML framework combining CMR imaging and clinical features for predicting MACEs in HCM patients. This framework demonstrated strong performance in both internal and external validation, with AUC values of 0.830 and 0.812, respectively, significantly outperforming the classical HCM Risk-SCD model [9].

Notably, AF is comorbid in 20–30% of HCM patients [48, 49]. Its development is closely linked to pathological mechanisms such as elevated left ventricular filling pressures, diastolic dysfunction, and outflow tract obstruction. However, the predictive capability of conventional HCM-AF scoring systems remains limited. Consequently, more precise tools are needed to forecast new-onset AF in HCM patients. In a prospective, multicenter cohort study, Lu *et al*. [37] utilized clinically significant variables such as left atrial volumes and diameters, as well as the difference in LVOTO pressure steps under resting and exercise loads to develop an ML model, which demonstrated sensitivity and specificity exceeding those of traditional scoring systems for predicting new-onset AF in HCM.

These breakthroughs not only confirm the value of AI in complex cardiovascular risk assessment, but also indicate that continuously optimizing the deep fusion of imaging-derived histological features and clinical big data holds promise for establishing a multi-dimensional risk warning system spanning the entire HCM disease course. Such a system could provide a reference basis for personalized diagnosis and treatment.

## 7. Discussion

Despite the significant progress of AI in HCM, limitations in existing research continue to hinder clinical translation. A primary challenge arises from the inherent opacity of DL architectures, particularly CNN, which function as non-transparent “black box” systems [50]. This lack of interpretability complicates clinicians’ ability to validate the diagnostic logic or treatment recommendations generated by AI models, thereby eroding trust in their outputs. Furthermore, this opacity heightens patient concerns regarding potential diagnostic errors, potentially leading to doctor-patient conflicts. To address this issue, researchers have developed explainability methods such as Gradient-Weighted Class Activation Maps (Grad-CAM). These techniques enhance the interpretability of AI models by revealing the underlying logic behind predictions. They do this by highlighting key regions within the input data that are crucial to the model’s decision-making process.

Grad-CAM utilizes gradient information from the target class to generate visual heatmaps, intuitively marking key regions within images that influence model judgments. This explains AI decision logic, enabling physicians to understand how AI extracts features from images and makes determinations. The method offers broader architectural compatibility and supports high-resolution classification explanations through integration with fine-grained visualization techniques [51]. Originating from game theory concepts, the SHapley Additive exPlanations (SHAP) value quantifies each feature’s contribution to individual predictions by providing a specific numerical measure of its impact. This offers a consistent and interpretable metric for feature importance [52]. Additionally, Local Interpretable Model-agnostic Explanations (LIME) is a model-agnostic local explanation method. It generates random samples within the local neighborhood of input data and fits these samples with a simple interpretable model to derive feature contributions for specific samples. This addresses the question: “Why did the model predict this outcome for this sample?” These technologies not only enhance the transparency of the model’s decision-making process but also align its reasoning logic with clinical cognitive frameworks, thereby gradually building physicians’ trust in AI-assisted decision-making. While these explainability tools point the way forward, proponents argue that existing limitations should spur further technical refinements and strengthen clinician training in AI integration; however, the fundamental tension between algorithmic complexity and clinical interpretability remains unresolved [53].

Moreover, methodological limitations in model development pose additional barriers to adoption. Insufficient data diversity and representativeness during training can trigger model bias and overfitting [54]. When deployed across heterogeneous clinical populations, these shortcomings impair predictive validity and generalizability. The resultant performance degradation compromises diagnostic accuracy and diminishes the reliability of treatment recommendations, ultimately limiting the practical utility of AI in real-world healthcare settings.

Compounding these issues, the current scarcity of high-quality, multicenter HCM datasets impedes comparative analysis of model performance across different patient cohorts and undermines the cumulative knowledge framework essential for iterative algorithm optimization [55].

As shown in recent work, Panichella *et al*. (2025) [56] provided an in-depth review of the transformative potential and practical challenges of artificial intelligence in the field of HCM. The authors critically note that while AI demonstrates significant capabilities in enhancing diagnostic accuracy, risk stratification, and treatment personalization through multimodal data integration, its clinical translation remains constrained by several fundamental barriers. These include the scarcity of high-quality, multicenter datasets, insufficient model interpretability, and the absence of standardized regulatory frameworks. Notably, the review highlights emerging technologies like digital twins and computer simulation trials (SMASH-HCM), which simulate disease progression and treatment responses by integrating biophysical models with patient-specific data.

Looking ahead, the future breakthroughs depend on establishing compliant data-sharing mechanisms, enhancing model transparency, and fostering interdisciplinary collaboration to bridge the gap between computational innovation and clinical practice. These initiatives will accelerate the development of clinically optimized AI systems, ultimately accelerating the translation of AI innovations into tangible clinical benefits for the diagnosis, risk stratification, and management of HCM.

## 8. Conclusion

AI can assist in identifying early lesion features in HCM, quantifying myocardial parameters, and predicting the risk of adverse cardiovascular events, thereby guiding the development of individualized treatment strategies. Numerous studies highlight the potential of AI algorithms in aiding clinical examinations (e.g., ECG, echocardiography, CMR). However, heterogeneity in patient data, model interpretability challenges, and data privacy concerns remain core issues requiring resolution. In the future, advances in multimodal data fusion, interpretable AI, and real-time monitoring technologies are expected to enable full-cycle intelligent management of HCM. Ultimately, AI-driven precision medicine will provide HCM patients with more efficient and personalized health management solutions, significantly enhancing their quality of life and long-term prognosis.
